# Novel oral anticoagulant use in adults with congenital heart disease: a single-center experience report

**DOI:** 10.1186/s43044-022-00326-1

**Published:** 2023-01-10

**Authors:** Daniel Samarai, Nazim Isma, Sandra Lindstedt, Joanna Hlebowicz

**Affiliations:** 1 Clinical Sciences, Lund University, Lund, Sweden; 2 Department of Cardiology, Skåne University Hospital, Lund University, Entrégatan 7, 221 85 Lund, Sweden; 3 Department of Cardiothoracic Surgery, Lund University, Lund University Hospital, Lund, Sweden

**Keywords:** Atrial fibrillation, Congenital heart disease, Novel oral anticoagulants

## Abstract

**Background:**

Adults with congenital heart disease (ACHD) are a group with an increased risk of thromboembolic complications and arrhythmias. Vitamin K antagonists are the most commonly used thromboprophylaxis therapy in this population. Studies on the efficacy and safety of novel oral anticoagulants (NOAC) are scare in ACHD. A retrospective study on ACHD patients on NOAC treatment registered in the National Quality Registry for Congenital Heart Disease, SWEDCON, and National Quality Registry for Atrial fibrillation and Anticoagulation, AuriculA, from Southern Sweden.

**Results:**

Thirty patients who had been taking NOAC treatment for a minimum of 3 months were included. Their median age was 55 years (SD 17 years) and 57% were male. Median follow-up was 17 months (IQR: 10–41). Eliquis was the most used NOAC (47%). Median CHA_2_DS_2_-VASc score was 2 (IQR: 0–3) and HAS-BLED was 1 (IQR: 0–2). Complex ACHD was prevalent in 27% of the patients. No thromboembolic events were recorded; however, one major bleeding, unspecified, was reported during the total cumulative patient follow-up time of 64 years.

**Conclusions:**

The results of our study, although limited in size, suggest that NOAC appear safe and effective in ACHD patients. Further and larger studies on NOAC in ACHD patients are warranted.

## Background

Adults with congenital heart disease (ACHD) are a patient group with an increased risk of thromboembolic complications and arrhythmias [1, 2]. The patient group is often anatomically heterogenic, attributing the increased risk of thromboembolism to multiple factors. A sufficient anticoagulant therapy for thromboprophylaxis indication is, therefore, a fundamental and essential treatment. NOAC, non-vitamin K oral anticoagulants, including the direct thrombin inhibitor dabigatran and factor Xa inhibitors (apixaban, edoxaban, rivaroxaban) are emerging clinically in the general population; however, they are used in a restricted manner in adults with CHD due to limited data on safety and efficacy. Vitamin K antagonists (VKA) are therefore the treatment of choice in these patients. The quality of VKA treatment can vary over time, being influenced by the intensity of the treatment, measured as time in therapeutic range (TTR) and the variability of the international normalized ratio (INR). A suboptimal treatment, with a low TTR and high INR variability, has been shown to increase the risk of adverse events [3]. In our recently published study in ACHD patients we observed an average VKA treatment of good quality with a high TTR and low INR variability [4]. In the same study population, we found a low rate of thromboembolic events and major bleeding events [5].

In ACHD patients treated with NOAC, a systemic review reported a low annual rate of thromboembolic and major bleeding events [6]. However, evidence for the use of NOAC in ACHD is still lacking. AuriculA, created in 2006, is a Swedish national quality register for atrial fibrillation and anticoagulation, which includes patient characteristics, indications and complications. As data on safety and efficacy are scarce and of value in this patient group, our aim was to report our experience of NOAC in ACHD patients, including the incidence of thromboembolism and major bleeding events.

## Methods

The National Quality Registry for Congenital Heart Disease, SWEDCON, was used to identify all ACHD patients retrospectively who had a registered use of NOAC (*n* = 33) in the South Region of Sweden (Region Skåne). This study ranged from 01/01/2006 to 23/02/2017. A minimal therapy of 3 months was set as an inclusion criterion; one patient was excluded. Two patients were excluded for being duplicates. Gender, age, treatment indication, type of anticoagulant, data on thromboembolic events and major bleeding events, and dates of starting and finishing anticoagulation were provided by AuriculA. AuriculA is a Swedish national quality registry for atrial fibrillation (AF) and anticoagulation. The registry was founded in 2006. The registry is used for patient characteristics, follow-up, dosage control of warfarin, indications, concurrent illnesses, and complications. SWEDCON provided the main congenital heart defect diagnosis, comorbidities, and interventions. Medical records of the patients were also reviewed for diagnoses and events. The CHA_2_DS_2_-VASc composite score (Congestive heart failure, Hypertension, Age ≥ 75 years [Doubled], Diabetes, Stroke/transient ischemic attack/thromboembolism [Doubled]—Vascular disease, Age 65–74 years and Sex category [Female]) was used to estimate the thromboembolic risk and HAS-BLED scores for the calculation of atrial fibrillation stroke risk and major bleeding risk were calculated at the time of each NOAC initiation [7, 8]. The HAS-BLED score assigns 1 point for the presence of each of the following bleeding risk factors: hypertension (H), abnormal renal and/or liver function (A), previous stroke (S), bleeding history (B), labile INR (L), elderly (E) and concomitant drugs and/or alcohol excess (D) (8). Start and stop date of anticoagulation therapy in the patient’s medical record also determined the duration of therapy. Congenital heart defects were classified into simple, moderate, and complex according to guidelines [9].

Complications registered by AuriculA were according to ISTH (International Society on Thrombosis and Haemostasis) definitions for major bleeding and clinically verified arterial or venous thrombosis [10, 11]. Minor bleedings were not searched for due to unreliable accessibility in medical journals. The register data in regard to anticoagulation therapy retained was quality controlled by a second confirmation of medical journals within the current study. The major bleedings were divided into three locations: intracranial, gastrointestinal or other bleeds. The thromboembolic (TE) events were divided into stroke/transient ischemic attack (TIA) or peripheral TE [12].

Descriptive statistics were used to summarize characteristics. Results were presented as median with SD or interquartile range (IQR), percentages and 95% confidence intervals (CI). Calculations and analyses were performed using SPSS Statistics Version 25 and Microsoft Excel Version 15.41.

The study was approved by the Regional Ethical Review Board in Lund. All patients included in SWEDCON are in accordance with a waiver regarding written informed consent supported by the proper authorities. Written or verbal information are the only requirements on a national level. The study was approved by the Regional Ethics Review Board in Lund, Sweden (Dnr 2017/260). The investigation conforms to the principles outlined in the Declaration of Helsinki.

## Results

Thirty adults with congenital heart disease were identified with NOAC as the anticoagulation therapy. The median age was 55 years (SD 17 years) with a slight male predominance (57%) (Table 1). The most common congenital heart disease was atrial shunt lesion (patent foramen ovale, PFO and atrial septal defect, ASD) (n = 9), followed by Tetralogy of Fallot (n = 4), Transposition of the great arteries (n = 3), pulmonary stenosis (n = 3), Anomalous pulmonary venous drainage (n = 2), Ebstein anomaly (n = 1), bicuspid aortic valve (n = 1), aorta coarctation (n = 1), cor triatriatum (n = 1), congenitally corrected transposition (n = 1), tricuspid atresia (n = 1), pulmonary atresia (n = 1) and congenital AV block (n = 1). The predominant indication was non-valvular atrial fibrillation (n = 26, 87%). Three patients with shunt lesions had indication ischemic stroke (PFO, ASD with transposition of the great arteries and ASD with congenitally corrected transposition). One patient with an ASD had a venous thromboembolism indication. Three patients had a bioprosthetic pulmonic valves, and two hade bioprosthetic mitral valves Edwards Magna II 27 and 29. In two patients with bioprosthetic valves the indication was atrial fibrillation and in one patient with a bioprosthetic valve the indication was ischemic stroke.

**Table 1:** Demographics, severity of congenital heart disease (CHD), comorbidities and results of CHA_2_DS_2_-VASc and HAS-BLED scores

Demographics	
All patients (n)	30
Gender	17 male (57%): 13 female (43%)
Median age	55 years (SD 17 years)
Median duration of therapy (months)	17 (min–max: 3–71)
Total cumulative duration of therapy (months)	764
*Complications*	
Events during therapy	1
Thrombotic or thromboembolic events	0
Bleeding events	1
*Severity of CHD*	
Simple	9
Moderate	11
Complex	8 (27%)
Unclassified	2
Defect repaired	27 (90%)
Bioprosthetic valves	5
*Indication for anticoagulation*	
Non-valvular atrial fibrillation	26
Venous thromboembolism	1
Cerebral embolism	3
*Comorbidities*	
Hypertension	9 (30%)
Diabetes	4 (13%)
*Type of novel oral anticoagulants (NOAC)*	
Apixaban	14
Rivaroxaban	9
Dabigatran	7
Scores	
Median CHA_2_DS_2_-VASc	2 (IQR: 0–3)
0	8
1	4
2	6
3	10
4	1
5	1
Median HAS-BLED	1 (IQR: 0–2)
0	13
1	4
2	12
3	1

Apixaban was the most commonly used NOAC (47%) followed by rivaroxaban (30%) and dabigatran (23%). Severity of the defects was predominantly simple-moderate (67%) with 27% being complex. Hypertension was the most common comorbidity (30%). Most of the patients (n = 25) were previously treated with warfarin and the treating physicians did choose to transition from warfarin to one of the new anticoagulants.

Median CHA_2_DS_2_-VASc was 2 points (IQR: 0–3) with 12 patients (40%) having a score higher than the median. The median HAS-BLED score was 1 point (IQR: 0–2), with 57% having a score lower than 2 points. In a total cumulative duration of 63 years of follow-up, no thromboembolic events were noted. One major bleeding event was recorded, described as “other,” not intracranial or gastric, giving an annual rate of 1.58 (95% CI: 0.08–7.78). The patient concerned was a female, 46 years of age, with Fallot’s tetralogy (moderate CHD) who was treated with rivaroxaban. She had a CHA_2_DS_2_-VASc score of 1 and HAS-BLED 0. The bleeding occurred after 19 months of treatment.

The most common cause of discontinued treatment was according to plan (16.7%), followed by a new indication (6.7%) and change to another anticoagulation (6.7%) (Table 2).

**Table 2:** Causes for discontinued novel oral anticoagulants (NOAC) treatment

Causes	Number (n)
According to plan	5
Due to bleeding	1
Due to other diseases	1
Due to poor compliance	1
Due to new indication	2
Change to other anticoagulation	2

## Discussion

This retrospective study reports a single-center experience of NOAC use in ACHD patients and found no thromboembolic and one major bleeding events during a median duration of 17 months of therapy.

Studies of NOAC use in ACHD patients are emerging but limited. A systemic review of NOAC use in ACHD patients included three studies with a total number of 766 patients [6]. The annual rate of thromboembolic and major bleeding events was 0.98% (95% CI: 0.51–1.86) and 1.74% (95% CI: 0.86–3.49), respectively. One study included Fontan patients only, reporting a higher annual rate of both events, confirming the increased risk of thromboembolism in ACHD patients of complex severity [13]. The two largest studies included 530 and 215 patients, not Fontan-exclusive [14, 15]. In these studies, the rate of complex severity of ACHD was about 40% and six and two thromboembolic events were registered, respectively. The total patient-years of follow-up was 896.3 years. Compared to our total patient-years of follow-up of 63.7 years, the low incidence of adverse events in our studies is plausible. However, compared to the largest included study of 530 patients, the median follow-up was 1 year, compared to 17 months in ours [16]. Furthermore, the prevalence of complex ACHD severity, which is a major risk factor for thromboembolism, was only 27% [13]. CHA_2_DS_2_-VASc > 2 was between 46 and 49% comparable to 40% in our study. For HAS-BLED, the scoring differed, with our study having 57% < 2 points and the other two studies reporting a higher 87–95% < 2 points [14, 15]. The risk of bleeding could thus be described to be higher in our study group, but the thromboembolic risk was lower with regard to the prevalence of complex severity defects and CHA_2_DS_2_-VASc scores. In one recent study, UNIVERSE Study evaluated the efficacy and safety of a rivaroxaban versus acetylsalicylic acid in 112 children during a high‐risk period early after Fontan procedure [17]. The study period was 12 months. Nonmajor bleeding occurred in 6% of the Fontan patients on rivaroxaban versus 9% on acetylsalicylic acid. The thromboembolic overall event rate was 2% in the rivaroxaban group and 9% in the acetylsalicylic acid group. The study showed a low prevalence of both thrombotic and bleeding events in the rivaroxaban and the acetylsalicylic acid groups. The above-mentioned factors together with the lower patient-years of follow-up could be an explanation for the low rates of thromboembolism and bleeding.

The incidence of thromboembolism and major bleeding events in ACHD with good quality VKA therapy was reported in our Swedish study with 213 patients from the same center: 1.0 (95%CI: 0.6–1.6) and 1.4 (95%CI: 0.9–2.2), respectively. In this study, the median duration of therapy was 6.6 years (± 3.3 years) [4, 5]. The follow-up time was longer and could, in our study, be a reason for an underestimated incidence of events. Risk factors, such as female gender, lower age, heart failure and history of thromboembolism, for insufficient anticoagulation and complications (thromboembolism and major bleeding) in ACHD patients with VKA therapy could be reasons to consider NOAC as an alternative [4, 5]. As heart failure is one of the strongest predictors for stroke, an increased risk of renal failure in these patients may make VKAs a more plausible choice [1]. NOAC was mostly discontinued according to plan, a new indication and change to another anticoagulant. Bleeding, other diseases and poor compliance were other less common causes for stopping NOAC therapy. As ACHD patients interact with health care at a younger age, compliance has been problematic. NOAC therapy can be an advantage in this patient group, especially for younger patients with normal heart and kidney function. NOAC can also be advantageous in patients on polypharmacy, which can interact with VKA. The benefits of NOAC, such as less regular blood tests, changing of dosing and consideration of food intake, could possibly appeal to younger patients and replace a potentially low compliance VKA therapy with its increased risk of thromboembolic events.

When comparing effectiveness and safety of NOAC to VKA for atrial fibrillation and venous thromboembolism, a systemic review and meta-analyses concluded NOAC to be comparable or superior to VKA in a non-ACHD population [18]. The study suggested individualizing the choice of anticoagulation therapy based on the benefit and safety patient profiles and characteristics. Use of NOAC in ACHD patients is emerging with data showing effectiveness and safety, although with caution as studies are rather small and study populations are typically heterogenic. NOAC may thus seem like a practical anticoagulation alternative when patient profiles and characteristics are associated with insufficient anticoagulation and complications.

## Conclusions

The results of our study, although limited in size, suggest that NOAC appear safe and effective in ACHD patients without mechanical valve protheses. Further and larger studies on NOAC in ACHD patients are warranted.

## Limitations

There are several limitations in this study that need to be acknowledged. The results and design of this retrospective study is descriptive and do not have a control group for comparison. The monitoring time did not cover the first prescribed period for every patient, and thus different exposure times exist for each patient. Adherence is unknown and difficult to evaluate. The study consists of a small heterogeneous ACHD population and short follow-up. Patients predominately simple-moderate severity of defects. This can influence treatment effect. Due to small number of events, we were no able to perform multivariate analysis for potential risk factors. Thus, the complications were thoroughly investigated and validated in the medical charts by the first author.

## Data Availability

Data may be available on request.

## Abbreviations

ACHD: Adults with congenital heart disease
AF: Atrial fibrillation
ASD: Atrial septal defect
INR: International normalized ratio
PFO: Patent foramen ovale
TE: Thromboembolic
TIA: Transient ischemic attack
TTR: Therapeutic range
NOAC: Novel oral anticoagulants
VKA: Vitamin K antagonists
